# Breeders’ knowledge on cattle fodder species preference in rangelands of Benin

**DOI:** 10.1186/s13002-018-0264-1

**Published:** 2018-11-08

**Authors:** Jéronime Marie-Ange Sènami Ouachinou, Gbèwonmèdéa Hospice Dassou, Akomian Fortuné Azihou, Aristide Cossi Adomou, Hounnankpon Yédomonhan

**Affiliations:** 1 Laboratory of Botany and Plant Ecology, Faculty of Sciences and Techniques, University of Abomey-Calavi, 01 BP 4521 Cotonou, Benin; 2 Laboratory of Applied Ecology, Faculty of Agronomic Sciences, University of Abomey-Calavi, 01 BP 526 Cotonou, Benin

**Keywords:** Cattle fodder species, Indigenous knowledge, Pasture walk, Top priority, Benin

## Abstract

**Background:**

We undertook ethnobotanical and ecological studies on fodder plants grazed by cattle across Benin national area. The study aims to ascertain the top priority fodder plants in order to catalogue the indigenous knowledge regarding their use.

**Methods:**

Data were collected through semi-structured interviews and covered 690 breeders and 40 days of pasture walk. These were analysed using similarity index of Jaccard (IS), relative frequency citation (RFC) and fodder value during pasture walk (FVPW).

**Results:**

We documented a total of 257 fodder plant species, of which 116 recorded during ethnobotanical investigations and 195 during pasture walk. These species belong to 181 genera and 54 families. Both methods shared 52 species. Leaves (58%) and leafy stem (28%) were the most grazed parts of plant. The most common species used as fodder included *Andropogon gayanus*, *Panicum maximum*, *Pterocarpus erinaceus* and *Flueggea virosa*. The top species with a highest FVPW were *Panicum maximum* and *Pterocarpus erinaceus*. A total of 16 species were considered as top fodder plants in Benin.

**Conclusions:**

The wide diversity of plants reported indicates that there is a number of promising fodder species in the flora of Benin. The insight gained in this study relating to bovine feeds could guide in the selection and introduction of feed innovations that could improve livestock production.

## Background

Worldwide, indigenous knowledge about the uses of plants as fodder or medicine played an important role in animal breeding development. Animal breeding is an ancient practice that represents an important subsistence source for low-income households worldwide [1]. In Benin, this activity plays an important role in the local economy and contributes to maintaining rural areas’ activity, their involvement in environment’s quality and poverty alleviation [2]. The considerable headway made in the field during recent decades, in particular the respect of schedules of vaccination campaigns becoming more and more rigorous, breeder awareness and their training on alimentation and the sanitary security of their cattle, and the increase of the credits allocated to them, have led to the steady growth of livestock production. From 1994 to 2013, livestock inventory in Benin increased by 39.18% for cattle and 35.40% for sheep and goats according to the FAOSTAT official database (http://www.fao.org/faostat/en/#home). Unfortunately, livestock sub-sector is still confronted by feeding problems [3], related to the availability and the quality of fodder resources. Indeed, natural pastures constitute the basis and, in most cases, the total food resources of ruminants. These pastures are in the majority dominated by annual plant species, characterised by a short development cycle that entirely unrolls in rain season. In this period, pasture contributes to ensure feed of cattle, but during the dry season, the longest season, it exists only the straws which are qualitatively poor and quantitatively deficient [4]. Although Benin is characterised by a vegetation type diversity [5], environmental pressures and strong influence of climatic seasonality limit the productive and nutritional potential of the fodder resources and hinder to maintain flocks, especially during drought periods. So, many breeders devote oneself to the ligneous that dispose leaves and fruits with high protein contents.

To face the unfavourable situation to the breeding development, it is important to capitalise traditional knowledge about fodders. Understanding traditional knowledge of people will result in four major outputs: the database creation of fodder plants consumed by cattle, identification of their properties and optimisation of their uses. To these, we can add the selection of fodders with top priority in stock farming based on their use value. According to Nunes et al. [6], a combination of traditional and scientific knowledges could allow to optimise the selection of useful fodder plants.

Ethnobotanical investigations about ruminants fodder plants have been developed in African countries such as Ethiopia, Nigeria, Ghana and Uganda [7–10], and elsewhere in Asia, India and Mexico [6, 11–13]. In Benin, there is no overall documentation about the relative importance of these feeds to farmers, although some researchers reported on tree fodders or medicinal tree fodders browsed by ruminants on natural pasture in northern Benin [14–16]. This study aims to (i) document fodder plants of natural pastures and state farms in Benin, (ii) assess the local knowledge regarding their use and (iii) select the most important fodder plants. The results of this study will be used to provide a checklist of fodder resources for further anatomical investigation and a possible improvement of food diet in controlled stock farming in Benin.

## Methods

### Study area

**Fig. 1:**
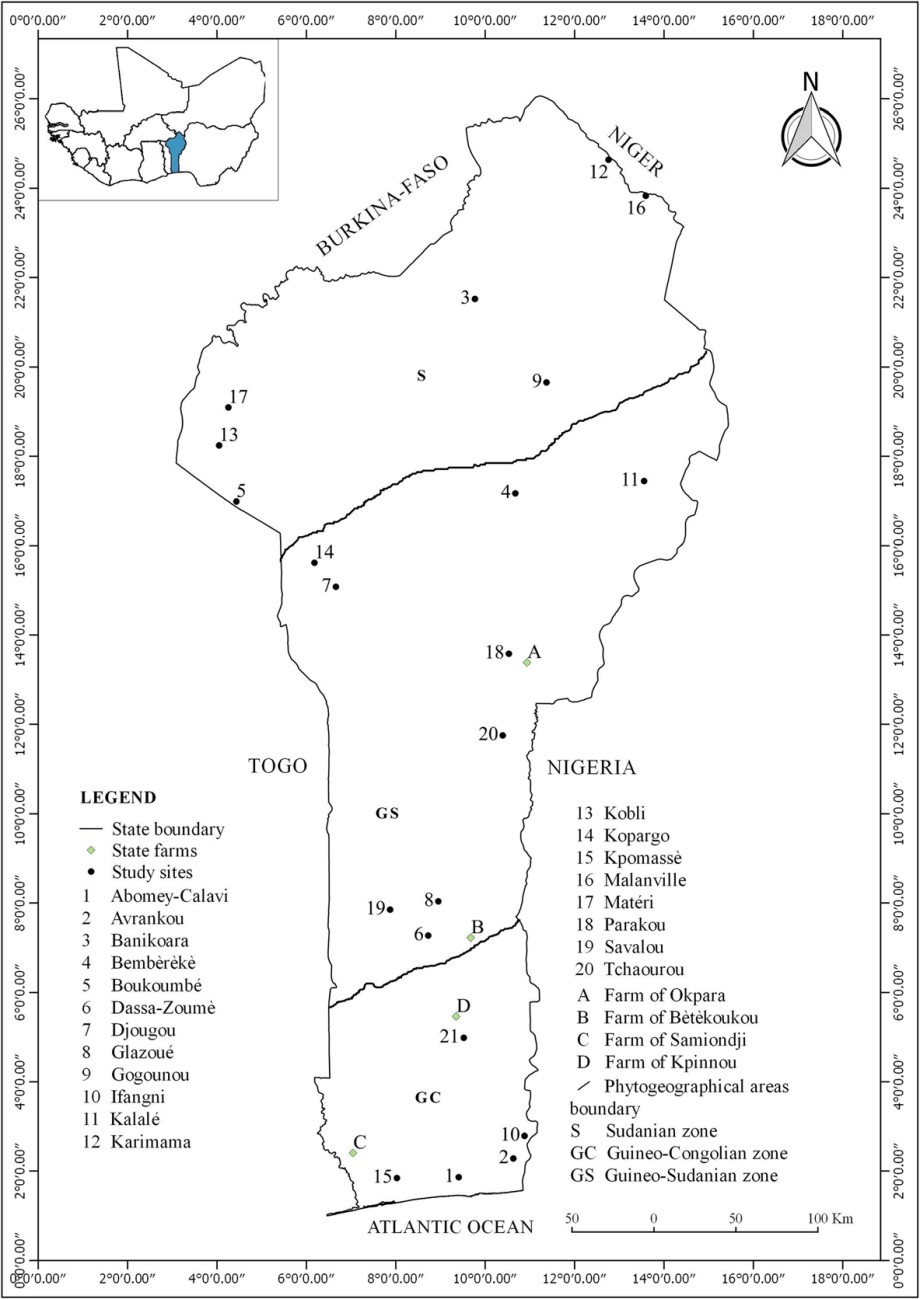
Location map of Benin with localities and farms covered by this study

Study was conducted across national area of the Republic of Benin (Fig. 1), located in West Africa between the latitudes 6° 10′ N and 12° 25′ N and longitudes 0° 45′ E and 3° 55′ E. It is bordered by Togo in the west, Nigeria in the east, Atlantic Ocean in the south and Burkina Faso and Niger in the north. The fieldwork was carried out in 23 localities (Fig. 1) and 4 state farms described in Table 1.

**Table 1:** Description of the state farms

State farms	Area (ha)	Climate zone	Annual rainfall (mm of rain)	Temp.	Soil	Vegetation	Breed type
FEK	380	Guinean	900–1100	29 °C	Ferralitic, clay-gravell	Small islands of forest, savannah	Girolando
FES	3600	Guinean	1123	27.6 °C	Clay	Savannah, forage plots	Lagunaire, Métis Azawak-lagunaire, Borgou
FEB	11,127	Sudano-guinean	900–1100	25 °C	Poorly evolved, ferruginous hydromorphic	Savannah, woodland, forest gallery	Borgou
FEO	33,000	Sudanian	1125	27 °C	Sandy, loamy	Woodland, savannah	Borgou, Girolando, Azawak

Source: MAEP [3]

*Temp.* temperature, *FEK* state farm of Kpinnou, *FES* state farm of Samiondji, *FEB* state farm of Bétécoucou, *FEO* state farm of Okpara

The study zone is submitted to three climate types (subequatorial in the southern zone, transition between subequatorial and tropical in the centre zone and tropical climate in the northern zone). The mean annual rainfall fluctuates from 900 to 1400 mm. The vegetation grows under three climate zones. According to Adomou [17], the southern zone consists of savannah, grassland, farmland and fallow intermingled with small islands of closed forest (semi-deciduous and swamp forests). In the centre and northern zones, the natural vegetation is essentially made of a patchwork of woodlands and savannahs with belts of riparian forest along rivers.

The national area contains 2807 plants species belonging to 1130 genera and 185 families [18]. The population of the country was estimated at 9,983,884 inhabitants with the majority involved in agriculture and breeding [19]. The livestock are mainly cattle (2,005,000), sheep and goats (2,413,000), pigs (293,200) and birds (15,900,000) [20]. The cattle production is concentrated at 85% in north of the country and largely dominates those of other animals [21]. The composition of cattle herds is characterised by a predominance of cows which expresses the dairy and breeding vocation that breeders give them. There are two general types of traditional cattle production in Benin: sedentary production in the Guinean region, which accounts for about 20% of the national herd, and transhumant production, which accounts for the other 80%. The exploitation of cattle is based on natural pastures and crop residues [22]. The Peulh own 95% of the national cattle herd and are thus the essential actors for the supply of animal proteins from the country [23].

### Data collection

We coupled ethnobotanical study and pasture walk for the data collection. During ethnobotanical investigations, 690 livestock owners were identified with the assistance of specialised animal production technicians for their experience in traditional breeding. Between February 2016 and May 2017, we conducted semi-structured individual interviews using 30 questionnaire slips per locality. The topics covered by the interview were socioeconomic parameters (ethnic group, sex, age, education level, profession, breeding type, size of livestock and source of knowledge) and fodder plants consumed by cattle (wild or crop, preference, parts of plants, collect modes and season of use).

In this paper, we use the term “fodder” to indicate plants grazed by the animals directly on pasture lands and those cut and carried to them. It includes grasses, cereal crops, legumes, shrubs and trees.

The pasture walk was authorised by the Coordinator of PAFILAV (Programme d’Appui aux Filières Lait et Viande) that ensures the management of state farms. It was conducted on the 4 state farms, and the data were recorded following the season (Table 2). On each farm, one herd and one animal were randomly selected by specialised animal production technician regarding state health of cattle. The pasture walk consisted of following the herd in natural vegetation neighbouring the farm between 9:00 am and 5:00 pm and to record plant species consumed by the targeted animal. The observations were repeated during 5 days.

**Table 2:** State farms and months of prospection

State farm	Dry months in 2016	Rainy months in 2017	Breed type
Kpinnou	January	June	Girolando
Samiondji	February	July	Lagunaire
Bétécoucou	March	September	Borgou
Okpara	April	June	Borgou

### Data analysis

#### Assessment of the taxonomical diversity

The data were organised, summarised and analysed using Excel spreadsheets. All species cited by informants and those recorded during pasture walk were identified using the Analytic Flora of Benin [18] and at the National Herbarium of Benin by comparing with already identified herbarium specimens. Voucher specimens of these plants were kept at the National Herbarium. A value of genus coefficient (GC) was determined by dividing the total number of species over the number of genera. In this study, recorded fodder flora presents high genus diversity when GC ≥ 1. Therefore, when GC < 1, this denotes low genus diversity.

The similarity index of Jaccard (IS) was calculated, and the similarity in fodder species composition between the pasture walk and the survey was compared following Kent and Coker [24]. IS was calculated as follows:

$$ \mathrm{IS}=\frac{c}{a+b-c} $$

where, *a* is the number of species found only in rangelands, *b* is the number of species only cited in survey and *c* is the number of common species in pasture walk and survey. Finally, IS was multiplied by 100 to calculate the percentage similarity in species composition between pasture walk and survey.

#### Breeders’ knowledge assessment

The difference in richness of grazed species during the drought and rain seasons was found through the chi-square test. The relative frequency of citation (RFC) and percentage of fodder value during pasture walk (FVPW) of each species were calculated.

Relative frequency of citation (RFC) was determined by dividing the number of informants citing a fodder species (FC) by the total number of informants in the survey (*N*). RFC was calculated by the formula as described:

$$ \mathrm{RFC}=\frac{\mathrm{FC}}{N} $$

The FVPW corresponds to the number of times a species was grazed by the target animal bovine during pasture walk.

A regression procedure was used to examine the correlation between RFC and FVPW. The Pearson correlation coefficient was used for this. Species present on rangelands and having RFC values higher than the mean of RFC were considered as priorities among fodder plants consumed by cattle in Benin.

## Results

### Taxonomical, morphological and habitat’s diversity of recorded fodder plants

A total of 257 fodder plants of which 116 for ethnobotanical investigations and 195 for pasture walk, with 52 common species, were recorded as consumed by cattle in Benin. These belong to 181 genera and 54 families. The average number of species recorded per family was 4.78, with 8 families (14.61% of the total) having more species than the average (Table 3). The 10 families that contributed 72.86% of all species were Leguminosae, Poaceae, Euphorbiaceae, Combretaceae, Asteraceae, Rubiaceae, Malvaceae, Moraceae, Acanthaceae and Amaranthaceae. The most speciose ones being Leguminosae (76 species, 29.45%) and Poaceae (57 species, 22.09%). These families were followed by Euphorbiaceae (12 species, 4.65%), Combretaceae (9 species, 3.48%), Asteraceae (9 species, 3.48%), Rubiaceae (7 species, 2.71%) and Malvaceae and Moraceae (6 species each, 2.37%). Twenty-seven families (50% of the total) were represented by only 1 species each. The remaining 27 families contributed between 2 and 5 species each (0.77–29.45% of the total). The ratio of the number of genera to the number of species was 1.41; we concluded high genera diversity among recorded species. The richest genera were *Desmodium*, *Hypparhenia* and *Indigofera* with 6 species each. The next most diversified genera in terms of species richness were *Andropogon*, *Crotalaria* (5 species each), *Combretum*, *Ficus*, *Schizachyrium*, *Senna* and *Tephrosia* (4 species each) followed by *Acacia*, *Albizia*, *Brachiaria*, *Commelina*, *Pennisetum*, *Setaria*, *Sida*, *Terminalia* and *Vigna* (3 species each). The low value of Jaccard’s similarity index (34%) means that the species harvested on pasture are distinct from those indicated by the breeders.

**Table 3:** Checklist of fodder plant species consumed by cattle in Benin

Family	Species (voucher number)	FVPW	Palatability	Lifespan	RFC	LF	PP	Season	Status
Acanthaceae	*Asystasia gangetica* (L.) T. Anderson (MAS 937)	6	*	Per	–	Herb	LS	D	W
	*Justicia flava* (Forssk.) Vahl (MAS 935)	6	***	Per	–	Herb	LS	D	W
	*Monechma ciliatum* (Jacq.) Milne-Redh. (MAS 603)	13	**	Ann	0.98	Herb	LS	D	W
	*Nelsonia canescens* (Lam.) Spreng. (MAS 1074)	13	**	Ann	–	Liana	LS	DR	W
Amaranthaceae	*Alternanthera sessilis* (L.) R.Br. ex Roth (MAS 1502)	0	–	Per	0.87	Liana	LS	D	W
	*Amaranthus spinosus* L. (MAS 275)	6	**	Ann	–	Herb	LS	D	W
	*Celosia argentea* L. (MAS 102)	25	**	Ann	–	Herb	Le	R	W
	*Pupalia lappacea* (L.) Juss. (MAS 551)	13	**	Per	–	Herb	LS	DR	W
Anacardiaceae	*Anacardium occidentale* L. (MAS 288)	0	–	Per	0.57	Shrub	Le	D	WC
	*Lannea acida* A.Rich. s.l. (MAS 1010)	3	*	Per	0.41	Tree	Le	D	W
	*Mangifera indica* L.	19	**	Per	–	Tree	Le, Fr	D	W
Annonaceae	*Annona senegalensis* Pers. (MAS 196)	9	*	Per	2.21	Shrub	Le	D	W
Araliaceae	*Cussonia arborea* Hochst. ex A. Rich. (MAS 344)	6	*	Per	0.39	Tree	Le	D	W
Arecaceae	*Elaeis guineensis* Jacq.	3	*	Per	–	Tree	Le	DR	C
Asclepiadaceae	*Periploca nigrescens* Afzel. (MAS 297)	6	**	Per	–	Liana	LS	DR	W
Asparagaceae	*Asparagus africanus* Lam. (MAS 49)	3	*	Ann	–	Herb	LS	R	W
Asteraceae	*Acanthospermum hispidum* DC. (MAS 181)	0	–	Ann	1.23	Herb	LS	R	W
	*Ageratum conyzoides* L. (MAS 127)	0	–	Ann	0.28	Herb	LS	D	W
	*Aspilia africana* (Pers.) Adams (MAS 42)	6	*	Per	–	Herb	LS	R	W
	*Aspilia bussei* O.Hoffm. & Muschl. (MAS 793)	0	–	Per	0.39	Herb	LS	DR	W
	*Aspilia helianthoides* (Schumach. & Thonn.) Olïv. & Diern (MAS 173)	9	*	Ann	–	Herb	LS	DR	W
	*Chromolaena odorata* (L.) R.M.King (MAS 96)	22	*	Per	–	Herb	LS	DR	W
	*Launaea taraxacifolia* (Willd.) Amin ex C.Jeffrey (MAS 828)	6	**	Ann	–	Herb	LS	DR	WC
	*Tridax procumbens* L. (MAS 818)	19	**	viv	0.90	Herb	LS	DR	W
	*Vernonia colorata* (WilId.) Drake (MAS 265)	6	*	Ann	–	Shrub	Le	D	W
Bignoniaceae	*Newbouldia laevis* (P.Beauv.) Seemann ex Bureau (MAS 62)	3	*	Ann	–	Shrub	Le	DR	W
Bignoniaceae	*Stereospermum kunthianum* Cham. (MAS 454)	3	**	Per	0.39	Tree	Le	D	W
Bombacaceae	*Adansonia digitata* L. (MAS 176)	0	–	Per	1.23	Tree	Le	DR	W
	*Bombax costatum* Pellegr. & Vuillet (MAS 167)	0	–	Per	0.26	Tree	Le	D	W
Capparaceae	*Cleome viscosa* L. (MAS 892)	9	*	Ann	0.39	Herb	LS	R	W
Celastraceae	*Gymnosporia senegalensis* (Lam.) Loes. (MAS 1038)	13	*	Per	–	Shrub	LS	D	W
Chrysobalanaceae	*Parinari curatellifolia* Planch. ex Benth. (MAS 487)	0	–	Per	0.64	Shrub	Le, Fr	DR	W
Cochlospermaceae	*Cochlospermum planchoni* Hook.f. (MAS 301)	22	**	Ann	–	Herb	Le, Fr	R	W
	*Cochlospermum tinctorium* A.Rich. (MAS 875)	9	*	Ann	–	Herb	Le	DR	W
Combretaceae	*Anogeissus leiocarpa* (De.) Guill. & Perr. (MAS 640)	25	**	Per	3.16	Tree	Le	D	W
	*Combretum collinum* Fresen. (MAS 789)	0	–	Per	0.77	Tree	Le	R	W
	*Combretum mucronatum* Schumach. & Thonn. (MAS 302)	16	**	Per	–	Liana	LS	D	W
	*Combretum nigricans* Lepr. ex Guill. & Perr. (MAS 1221)	0	–	Per	1.08	Tree	Le	D	W
	*Combretum paniculatum* Vent. (MAS 593)	3	*	Per	–	Liana	LS	DR	W
	*Pteleopsis suberosa* Engl. & Diels (MAS 700)	13	**	Per	–	Shrub	Le	R	W
	*Terminalia avicennioides* Guill. & Perr. (MAS 696)	6	*	Per	0.51	Shrub	Le	D	W
	*Terminalia laxiflora* Engl. (MAS 1390)	3	*	Per	–	Shrub	Le	D	W
	*Terminalia macroptera* Guill. & Perr. (MAS 229)	3	*	Per	0.13	Shrub	Le	DR	W
Commelinaceae	*Commelina benghalensis* L. (MAS 52)	0	–	Per	0.64	Herb	WP	D	W
	*Commelina erecta* L. (MAS 79)	9	**	Per	–	Herb	LS	R	W
	*Commelina forskalaei* Vahl (MAS 177)	0	–	Per	0.15	Herb	WP	R	W
Connaraceae	*Rourea coccinea* (Thonn. ex Schumach.) Benth. (MAS 15)	19	**	Ann	–	Shrub	LS	DR	W
Convolvulaceae	*Hewittia scandes* (Milne) Mabberley (MAS 884)	25	*	Per	–	Herb	LS	D	W
	*Ipomoea involucrata* P. Beauv. (MAS 917)	6	**	Ann	–	Herb	LS	D	W
	*Merremia pinnata* (Hochst. ex Choisy) Hallier (MAS 553)	12	*	Ann	–	Herb	LS	R	W
Cucurbitaceae	*Momordica charantia* L. (MAS 1052)	0	–	Per	0.64	Liana	LS	D	W
Cyperaceae	*Cyperus difformis* L. (MAS 738)	3	*	Ann	–	Herb	WP	D	W
	*Cyperus rotundus* L. (MAS 430)	1	*	Per	–	Herb	Le	DR	W
Cyperaceae	*Cyperus sphacelatus* L. (MAS 550)	0	–	Ann	0.46	Herb	WP	R	W
Discoreaceae	*Dioscorea cayenensis* Lam. (MAS 146)	3	*	Ann	–	Herb	Le	DR	WC
Ebenaceae	*Diospyros mespiliformis* Hochst. ex A.DC. (MAS 628)	0	–	Per	0.31	Tree	Le	D	W
Euphorbiaceae	*Alchornea cordifolia* (Schumach. & Thonn.) Müll.Arg. (MAS 915)	6	*	Per	–	Shrub	Le	D	W
	*Antidesma venosum* E.Mey. ex Tul. (MAS 386)	13	*	Per	–	Shrub	Le	D	W
Euphorbiaceae	*Bridelia ferruginea* Benth. (MAS 180)	19	**	Per	1.16	Shrub	Le, Fr	D	W
	*Euphorbia convolvuloides* Hochst. ex Benth. (MAS 446)	13	*	Ann	–	Herb	LS	R	W
	*Flueggea virosa* (Roxb. ex Willd.) Voigt (MAS 607)	47	***	Per	5.14	Shrub	LS	D	W
	*Hymenocardia acida* Tul. (MAS 815)	13	**	Per	0.26	Shrub	Le	DR	W
	*Jatropha gossypiifolia* L. (MAS 330)	3	*	Per	–	Shrub	LS	D	W
	*Mallotus oppositifolius* (Geisel.) Müll.Arg. (MAS 254)	6	**	Per	0.77	Shrub	LS	D	W
	*Manihot esculenta* Crantz	13	**	Per	0.31	Shrub	Le, tub	D	C
	*Margaritaria discoidea* (Baill.) Webster (MAS 292)	9	*	Per	–	Tree	Le	DR	W
	*Phyllanthus amarus* Schumach. & Thonn. (MAS 184)	31	**	Per	–	Herb	LS	D	W
	*Phyllanthus muellerianus* (Kuntze) Exell (MAS 233)	19	**	Ann	1.08	Liana	LS	DR	W
Flacourtiaceae	*Flacourtia indica* (Burm.f.) Merr. (MAS 212)	6	*	Per	–	Shrub	Le	D	W
Lamiaceae	*Hyptis suaveolens* (L.) Poit. (MAS 541)	6	*	Ann	0.62	Herb	LS, Fl	R	W
	*Leucas martinicensis* (Jacq.) R.Br. (MAS 502)	6	*	Ann	–	Herb	LS, Fl	R	W
Leg-Caesalpinioideae	*Afzelia africana* Sm. (MAS 162)	16	***	Per	1.59	Herb	Le	DR	W
	*Burkea africana* Hook. (MAS 163)	0	–	Per	0.41	Tree	Le	DR	W
	*Cassia sieberiana* DC. (MAS 209)	0	–	Per	0.77	Shrub	LS	R	W
	*Chamaecrista mimosoides* (L.) Greene (MAS 258)	9	*	Ann	–	Herb	LS	R	W
	*Chamaecrista rotundifolia* (Pers.) Greene (MAS 416)	16	**	Ann	0.51	Herb	WP	D	W
	*Daniellia oliveri* (Rolfe) Hutch. & Dalziel (MAS 123)	0	–	Per	1.34	Tree	Le, Fl, Fr	D	W
	*Detarium microcarpum* Guill. & Perr. (MAS 218)	6	**	Per	1.44	Tree	LS	R	W
	*Dialium guineense* WiIld. (MAS 1045)	3	*	Per	–	Tree	Le	DR	W
	*Isoberlinia doka* Craib & Stapf (MAS 173)	0	–	Per	0.28	Tree	Le	R	W
	*Piliostigma thonningii* (Schumach.) Milne-Redh. (MAS 322)	31	**	Per	2.83	Tree	Le, Fr	D	W
	*Senna hirsuta* (L.) H.S. Irwin & Barneby (MAS 488)	6	**	Ann	–	Herb	LS	D	W
	*Senna obtusifolia* (L.) H.S.Irwin & Barneby (MAS 359)	3	*	Per	–	Herb	Le	R	W
	*Senna occidentalis* (L.) Link (MAS 812)	3	*	Ann	–	Herb	LS	R	W
	*Senna siamea* (Lam.) H.S.Irwin & Barneby (MAS 336)	9	**	Ann	–	Tree	Le	DR	W
Leg-Mimosoideae	*Acacia auriculiformis* A.Cunn. ex Benth. (MAS 27)	6	**	Per	–	Tree	Le	R	W
	*Acacia nilotica* (L.) Willd. (MAS 718)	3	*	Per	–	Tree	Le	D	W
Leg-Mimosoideae	*Acacia sieberiana* DC. (MAS 259)	13	**	Per	1.54	Tree	Le, Fr	DR	W
	*Albizia adianthifolia* (Schumach.) W.F. Wright (MAS 84)	3	*	Per	–	Tree	Le	D	W
	*Albizia lebbeck* (Schumach.) W.F. Wright (MAS 433)	6	*	Per	0.64	Tree	Le	D	W
	*Albizia zygia* (De.) J.F.Macbr. (MAS 1243)	3	*	Per	–	Tree	Le	D	W
	*Dichrostachys cinerea* (L.) Wight & Arn. (MAS 1319)	0	–	Per	0.39	Shrub	Le, Fr	DR	W
	*Entada africana* GuilI. & Perr. (MAS 226)	3	*	Per	0.39	Tree	Le	D	W
	*Leucaena leucocephala* (Lam.) De Wit (MAS 429)	22	***	Per	1.41	Tree	Le	D	WC
	*Mimosa pigra* L. (MAS 267)	6	**	Per	–	Shrub	Le	D	W
	*Parkia biglobosa* (Jacq.) R.Br. ex Benth. (MAS 752)	0	–	Per	0.90	Tree	Le	D	W
	*Pithecellobium dulce* (Roxb.) Benth. (MAS 1007)	3	*	Per	–	Tree	LS	D	W
	*Prosopis africana* (GuilI. & Perr.) Taub. (MAS 953)	31	***	Per	2.52	Tree	Le, Fl	R	W
Leg-Papilionoideae	*Aeschynomene americana* L. (MAS 141)	9	***	Per	–	Shrub	Le	R	W
	*Alysicarpus ovalifolius* (Schumach.) J.Léonard (MAS 711)	0	–	Per	1.16	Herb	LS	D	W
	*Alysicarpus rugosus* (Willd.) DC. (MAS 166)	6	**	Per	–	Herb	Le, Fl	DR	W
	*Arachis hypogea* L. (MAS 94)	0	–	Per	0.51	Herb	Le	DR	C
	*Calopogonium mucunoides* Desv. (MAS 112)	9	**	Per	–	Liana	LS	R	W
	*Centrosema pubescens* Benth. (MAS 295)	28	**	Per	0.64	Liana	LS	D	W
	*Crotalaria comosa* Baker (MAS 328)	3	*	Ann	–	Herb	LS	D	W
	*Crotalaria macrocalyx* Benth. (MAS 393)	0	–	Ann	0.77	Herb	LS, Fl	D	W
	*Crotalaria microcarpa* Hochst. ex Benth. (MAS 673)	0	–	Ann	0.90	Herb	LS	D	W
	*Crotalaria ononoides* Benth. (MAS 636)	3	*	Ann	–	Herb	LS	D	W
	*Crotalaria pallida* Aiton (MAS 109)	3	*	Ann	–	Herb	LS	D	W
	*Desmodium adscendens* (Sw.) DC. (MAS 617)	6	*	Per	–	Herb	LS	DR	W
	*Desmodium gangeticum* (L.) DC. (MAS 615)	6	*	Per	–	Shrub	Le	DR	W
	*Desmodium hirtum* Guin. & Perr. (MAS 326)	0	–	Ann	0.67	Herb	LS	D	W
	*Desmodium ramossissimum* D.Don (MAS 524)	3	*	Ann	–	Herb	Le	DR	W
	*Desmodium salicifolium* (Poir.) DC. (MAS 571)	0	–	Ann	0.80	Herb	LS	D	W
	*Desmodium velutinum* (Willd.) DC. (MAS 303)	25	**	Ann	0.77	Herb	LS	R	W
	*Eriosema griseum* Baker (MAS 631)	6	**	Per	–	Shrub	Le	R	W
	*Glycine max* (L.) Merr. (MAS 247)	0	–	Ann	0.41	Herb	Le	D	C
Leg-Papilionoideae	*Indigofera conjugata* Baker (MAS 921)	3	**	Per	–	Liana	LS	D	W
	*Indigofera dendroides* Jacq. (MAS 304)	6	**	Ann	0.77	Herb	LS	R	W
	*Indigofera hirsuta* L. (MAS 159)	6	*	Ann	–	Herb	Le, Fr	DR	W
	*Indigofera paniculata* Vahl ex Pers. (MAS 118)	0	–	Ann	0.39	Herb	LS, Fr	DR	W
	*Indigofera stenophylla* Guill. & Perr. var. stenophylla (MAS 573)	0	–	Ann	0.39	Herb	Le	D	W
	*Indigofera tinctoria* L. (MAS 806)	6	*	Per	–	Herb	LS	DR	W
	*Lonchocarpus sericeus* (Poir.) (MAS 363)	25	***	Per	0.90	Tree	Le	R	W
	*Millettia thonningii* (Schumach. & Thonn.) Baker (MAS 276)	3	*	Ann	–	Shrub	Le	DR	W
	*Pericopsis laxiflora* (Benth. ex Baker) Meeuwen (MAS 821)	6	*	Ann	–	Tree	Le	R	W
	*Philenoptera cyanescens* (Sehumacb. & Thonn.) Roberty (MAS 762)	0	–	Per	1.34	Shrub	Le	R	W
	*Philenoptera laxiflora* (Guill. & Perr.) Roberty (MAS 582)	0	–	Per	1.08	Tree	LS	D	W
	*Pseudarthria hookeri* Wight & Am. var. hookeri (MAS 21)	19	*	Per	–	Herb	LS	D	W
	*Pseudovigna argentea* (Willd.) Verdc. (MAS 541)	25	**	Per	–	Herb	LS	R	W
	*Pterocarpus erinaceus* Poir. (MAS 1012)	50	***	Per	5.35	Tree	Le	DR	W
	*Rhynchosia sublobata* (Sehumaeh. & Thonn.) Meikle (MAS 322)	6	**	Per	–	Herb	LS	DR	W
	*Sesbania grandiflora* (L.) Poir. (MAS 396)	25	*	Per	–	Shrub	Le	D	W
	*Sesbania pachycarpa* DC. ssp. pachycarpa (MAS 903)	9	**	Per	–	Herb	Le	DR	W
	*Stylosanthes fruticosa* (Retz.) Alston (MAS 669)	13	**	Per	–	Herb	LS	D	W
	*Stylosanthes hamata* (L.) Taub. (MAS 709)	3	*	Per	–	Herb	Le	DR	W
	*Swartzia madagascariensis* Desv. (MAS 1061)	3	**	Per	–	Tree	Le	D	W
	*Tephrosia bracteolata* Guilt. & Perr. (MAS 914)	16	*	Per	–	Herb	LS	DR	W
	*Tephrosia elegans* Schumach. (MAS 149)	3	**	Ann	–	Herb	LS	D	W
	*Tephrosia purpurea* (L.) (MAS 173)	13	**	Ann	1.54	Herb	LS	D	W
	*Tephrosia villosa* (L.) Pers. (MAS 1033)	13	**	Per	–	Herb	LS	D	W
	*Teramnus labialis* (L.f.) Spreng. (MAS 571)	3	*	Ann	–	Herb	Le	D	W
	*Vigna racemosa* (G.Don) Hutch. & Dalziel (MAS 249)	3	*	Per	–	Herb	Le	D	W
	*Vigna reticulata* Hook.f. (MAS 332)	3	*	Per	–	Herb	LS	DR	W
	*Vigna unguiculata* (L.) Walp. (MAS 989)	0	–	Ann	0.64	Herb	Le	DR	C
	*Zornia glochidiata* Rchb. ex DC. (MAS 963)	3	*	Ann	–	Herb	LS	DR	W
Loganiaceae	*Strychnos innocua* Delile (MAS 1053)	0	–	Ann	0.26	Shrub	Le	DR	W
Malvaceae	*Gossypium sp.* (MAS 753)	0	–	Ann	0.26	Herb	Le	R	C
	*Hibiscus asper* Hook.f. (MAS 1162)	13	*	Ann	0.57	Herb	Le, Fl	D	W
	*Sida acuta* Burm.f. (MAS 92)	25	**	Ann	0.64	Herb	LS	D	W
	*Sida garckeana* Pol. (MAS 173)	0	*	viv	0.57	Herb	LS	D	W
	*Sida linifolia* Juss. ex Cav. (MAS 33)	13	*	viv	–	Herb	Le	DR	W
Meliaceae	*Azadirachta indica* A.Juss. (MAS 1018)	19	**	Per	–	Tree	Le	D	W
	*Khaya senegalensis* (Desr.) A.Juss. (MAS 436)	0	–	Per	1.39	Tree	Le	R	W
	*Pseudocedrela kotschyii* (Schweinf.) Harms. (MAS 633)	31	**	Per	2.57	Tree	Le	D	W
Menispermaceae	*Cissampelos mucronata* A. Rich. (MAS 916)	9	**	Per	–	Liana	LS	D	W
Moraceae	*Antiaris toxicaria* Lesch. (MAS 402)	3	*	Per	–	Tree	Le	D	W
	*Ficus ingens* (Miq.) Miq. (MAS 113)	0	–	Per	0.26	Tree	Le	D	W
	*Ficus sur* Forssk. (MAS 77)	16	**	Per	–	Tree	LS	DR	W
	*Ficus sycomorus* L. (MAS 169)	0	–	Per	0.36	Tree	Le	D	W
	*Ficus variifolia* Warb. (MAS 412)	0	–	Per	0.31	Tree	Le	DR	W
Moringaceae	*Moringa oleifera* Lam. (MAS 761)	3	*	Per	–	Shrub	Le	DR	WC
Musaceae	*Musa sp*. L.	6	*	Per	–	Herb	Le	D	C
Myrtaceae	*Syzygium guineense* (WiIld.) DC. var. guineense (MAS 319)	3	*	Per	–	Tree	Le	D	W
Nyctaginaceae	*Boerhavia diffusa* L. (MAS 611)	6	**	Ann	–	Herb	WP	D	W
	*Boerhavia erecta* L. (MAS 96)	6	*	Ann	0.31	Herb	WP	D	W
Ochnaceae	*Lophira lanceolata* Tiegh. ex Keay (MAS 188)	9	**	Per	–	Tree	Le	D	W
Olacaceae	*Olax subscorpioidea* Oliv. (MAS 256)	6	*	Per	–	Shrub	Le, Fr	D	W
Opiliaceae	*Opilia amentacea* Roxb. (MAS 202)	6	*	Per	–	Liana	LS	D	W
Passifloraceae	*Passiflora foetida* L. (MAS 436)	13	**	Per	0.57	Herb	WP	D	W
Poaceae	*Acroceras amplectens* Stapf (MAS 22)	6	*	Ann	–	Herb	Le	DR	W
	*Anadelphia afzeliana* (Rendle) Stapf (MAS 306)	3	*	Per	–	Herb	Le	R	W
	*Andropogon chinensis* (Nees) Merr. (MAS 921)	3	*	Per	–	Herb	Le	DR	W
	*Andropogon fastigiatus* Sw. (MAS 88)	3	*	Ann	–	Herb	Le	D	W
	*Andropogon gayanus* Kunth (MAS 109)	47	**	Ann	5.81	Herb	Le	DR	WC
	*Andropogon schirensis* Rochst. ex A.Rich. (MAS 534)	13	**	Per	–	Herb	Le	DR	W
	*Andropogon tectorum* Schumach. & Thonn. (MAS 508)	31	**	Per	4.24	Herb	Le	R	W
Poaceae	*Aristida hordeaca* Kunth (MAS 1033)	9	**	Ann	–	Herb	Le	DR	W
	*Aristida kerstingii* Pilger (MAS 339)	3	**	Ann	–	Herb	Le	D	W
	*Bambusa vulgaris* Schrad. ex Wendel (MAS 1020)	0	–	Per	0.13	Tree	Le	R	W
	*Beckeropsis uniseta* (Nees) K.Schum. (MAS 1078)	0	–	Ann	0.33	Herb	Le	D	W
	*Brachiaria deflexa* (Schumach.) Robyns (MAS 1001)	6	*	Per	–	Herb	Le	D	W
	*Brachiaria mutica* (Forssk.) Stapf (MAS 444)	19	**	Per	–	Herb	WP	D	W
	*Brachiaria ruziziensis* Germain & Evrard (MAS 757)	13	*	Per	–	Herb	Le	D	W
	*Ctenium elegans* Kunth (MAS 43)	3	*	Ann	–	Herb	Le	D	W
	*Dactyloctenium aegyptium* (L.) Wild. (755)	9	**	Ann	–	Herb	Le	D	W
	*Digitaria horizontalis* Wild. (MAS 453)	13	**	Ann	2.29	Herb	Le	D	WC
	*Eleusine indica* Gaertn. (MAS 1073)	0	–	Ann	0.39	Herb	Le	D	W
	*Elionurus elegans* Kunth (MAS 523)	3	*	Ann	–	Herb	Le	D	W
	*Elymandra androphila* (Stapf) Stapf (MAS 771)	3	*	Per	–	Herb	Le	D	W
	*Eragrostis aspera* (Jacq.) Nees (MAS 343)	0	–	Ann	0.57	Herb	Le	D	W
	*Euclasta condylotricha* (Steud.) Stapf (MAS 1065)	0	–	Ann	0.26	Herb	Le	D	W
	*Heteropogon contortus* (L.) P.Beauv. (MAS 817)	0	–	Per	0.15	Herb	WP	D	W
	*Hypparhenia barteri* (Rack.) Stapf (MAS 117)	19	**	Ann	–	Herb	Le	R	W
	*Hypparhenia cyanescens* (Stapf) Stapf (MAS 943)	3	*	Per	–	Herb	Le	D	W
	*Hypparhenia involucrata* Stapf (MAS 418)	0	–	Ann	0.57	Herb	Le	DR	W
	*Hypparhenia mutica* Clayton (MAS 1017)	6	*	Per	–	Herb	Le	D	W
	*Hypparhenia rufa* (Nees) Stapf (MAS 713)	0	–	Per	0.64	Herb	Le	R	W
	*Hypparhenia subplumosa* Stapf (MAS 602)	3	*	Per	–	Herb	Le	D	W
	*Imperata cylindrica* (L.) P.Beauv. (MAS 337)	13	***	Per	1.16	Herb	WP	DR	W
	*Loudetia togoensis* (Pilg.) C.E.Hubbard (MAS 114)	3	*	Ann	–	Herb	Le	DR	W
	*Microchloa indica* (L.) P.Beauv. (MAS 504)	0	–	Ann	0.57	Herb	Le	D	W
	*Monocymbium ceresiiforme* (Nees) Stapf (MAS 1013)	8	***	Ann	–	Herb	Le	R	W
	*Oryza sativa* L. (MAS 203)	0	–	Ann	0.90	Herb	Le	R	C
	*Panicum maximum* Jacq. (MAS 93	50	***	Ann	5.45	Herb	Le	D	WC
	*Panicum repens* L. (MAS)	6	**	Per	–	Herb	Le	R	WC
	*Paspalum scrobiculatum* L. (MAS 104)	3	*	Per	–	Herb	Le	D	W
Poaceae	*Paspalum vaginatum* Sw. (MAS 26)	19	*	Per	0.31	Herb	Le	R	W
	*Pennisetum glaucum* (L.) R.Br. (MAS 710)	13	*	Ann	–	Herb	Le	R	W
	*Pennisetum pedicellatum* Trin. (MAS 309)	19	*	Ann	0.26	Herb	Le	D	W
	*Pennisetum polystachion* (L.) Schult. (MAS 421)	13	*	Ann	–	Herb	Le	D	W
	*Rottboellia cochinchinensis* (Lour.) (MAS 205)	13	*	Per	–	Herb	Le	R	W
	*Saccharum officinarum* L. (MAS 630)	0	–	Per	0.39	Herb	Le	R	WC
	*Schizachyrium brevifolium* (Sw.) Nees (MAS 208)	9	*	Per	–	Herb	Le	R	W
	*Schizachyrium platyphyllum* (Franch.) Stapf (MAS)	9	*	Ann	–	Herb	Le	DR	W
	*Schizachyrium ruderale* Clayton (MAS 501)	9	*	Per	–	Herb	Le	D	W
	*Schizachyrium sanguineum* (Retz.) Alston (MAS 1054)	9	*	Ann	–	Herb	Le	DR	W
	*Setaria gracilipes* C.E.Hubb. (MAS 129)	6	*	Ann	–	Herb	Le	D	W
	*Setaria megaphylla* (Steud.) T.Durand & Sehinz (MAS 401)	0	–	Ann	0.31	Herb	Le	R	W
	*Setaria pumila* (Poir.) Roem. & Schult. (MAS 308)	3	*	Per	–	Herb	Le	R	W
	*Sorghum bicolor* (L.) Moench (MAS 152)	0	–	Ann	0.39	Herb	Le	D	C
	*Sporobolus pyramidalis* P.Beauv. (MAS 1044)	3	*	Ann	0.67	Herb	Le	D	W
	*Stenotaphrum dimidiatum* (L.) Brongn. (MAS 142)	3	*	Per	–	Herb	Le	DR	W
	*Thelepogon elegans* Roth ex Roem. & Sehult. (MAS 744)	0	–	Per	0.41	Herb	Le	R	W
	*Tristachya superba* (De Not.) Schweinf. & Aschers. (MAS 519)	6	*	Ann	–	Herb	Le	R	W
	*Vetiveria nigritana* (Benth.) Stapf (MAS 1071)	0	–	Per	0.13	Herb	Le	D	W
	*Zea mays* L.	0	–	Ann	0.51	Herb	Le	D	C
Polygalaceae	*Securidaca longepedunculata* Fresen. (MAS 74)	9	*	Per	0.26	Herb	LS	DR	W
Pontederiaceae	*Eichhornia crassipes* (Mart.) SolmsLaub. (MAS 531)	3	**	Per	–	Herb	Le, Fl	D	W
Rubiaceae	*Gardenia ternifolia* Sehumaeh. & Thonn. (MAS 59)	16	**	Per	0.39	Tree	Le, Fr	DR	W
	*Mitracarpus hirtus* (L.) DC. (MAS 346)	13	*	Per	–	Herb	LS, Fl	D	W
	*Mitragyna inermis* (Willd.) Kuntze (MAS 153)	3	*	Ann	1.03	Tree	Le	R	W
Rubiaceae	*Morinda lucida* Benth. (MAS 75)	13	*	Per	–	Tree	Le	D	W
	*Sarcocephalus latifolius* (Sm.) E.A.Bruce (MAS 154)	25	**	Per	0.67	Shrub	Le	R	W
	*Spermacoce hepperrana* Verdc. (MAS 243)	9	*	Ann	–	Herb	Le	R	W
	*Spermacoce stachydea* DC. (MAS 617)	6	*	Ann	1.03	Herb	Le	R	W
Sapindaceae	*Blighia sapida* Konig (MAS 139)	6	**	Per	–	Tree	Le	DR	W
Sapindaceae	*Deinbollia pinnata* (Poir.) Schumach. & Thonn. (MAS 44)	13	*	Per	–	Shrub	LS	R	W
	*Paullinia pinnata* L. (MAS102)	25	**	Ann	–	Liana	LS	D	W
Sapotaceae	*Mimusops kummel* Bruce ex A.DC. (MAS 409)	19	**	Per	–	Shrub	Le	D	W
	*Pouteria alnifolia* (Baker) Roberty var. alnifolia (MAS 211)	6	*	Per	–	Shrub	Le	D	W
	*Vitellaria paradoxa* C.F.Gaertn. (MAS 312)	19	***	Per	1.03	Tree	Le	D	W
Scrophulariaceae	*Striga hermonthica* (DeliIe) Benth. (MAS 66)	0	–	Per	0.93	Herb	Le	DR	W
Solanaceae	*Harrisonia abyssinica* R.Br. ex A.Juss. (MAS 231)	6	*	Per	–	Shrub	Le	D	W
Sterculiaceae	*Sterculia setigera* Delile (MAS 321)	0	–	Per	0.64	Tree	Le	DR	W
	*Waltheria indica* L. (MAS 87)	0	–	Per	0.82	Herb	LS	R	W
Taccaceae	*Tacca leontopetaloides* (L.) Kuntze (MAS 545)	13	**	Per	–	Herb	LS	DR	W
Tiliaceae	*Grewia cissoides* Hutch. & DalzieI (MAS 273)	0	–	Per	0.46	Shrub	LS	D	W
	*Grewia villosa* Willd. (MAS 718)	6	*	Per	0.90	Shrub	Le	D	W
	*Triumfetta pentandra* A.Rich. (MAS 313)	0	–	Per	0,31	Herb	LS	R	W
Verbenaceae	*Clerodendrum capitatum* (WilId.) Schumach. & Thonn. (MAS 362)	19	*	Per	–	Liana	LS	D	W
	*Gmelina arborea* Roxb. (MAS 411)	19	***	Per	–	Tree	LS	D	W
	*Vitex doniana* Sweet (MAS 143)	0	–	Per	0.98	Tree	Le	D	W
Zingiberaceae	*Costus spectabilis* (Fenzl) K.Schum. (MAS 609)	6	**	Per	–	Herb	Le, Fl	D	W
	*Siphonochilus aethiopicus* (Schweinf.) B.L.Burtt (MAS 164)	19	*	Per	–	Herb	Le	D	W
Zygophyllaceae	*Balanites aegyptiaca* (L.) Delile (MAS 180)	0	–	Per	0.31	Shrub	Le	D	W
	*Tribulus terrestris* L. (MAS 201)	3	*	Ann	–	Herb	LS	DR	W

*Leg-* Leguminosae; *FVPW* fodder value during pasture walk; *RFC* relative citation frequency; lifespan (*Per* perennial, *Ann* annual); *PP* plant parts (*Le* leaves, *LS* leafy stems, *Fr* fruits, *Fl* flowers, *tub* tubercle, *WP* whole plant); status (*W* wild, *C* cultivated, *WC* wild and cultivated); palatability (*fairly palatable, **weakly palatable, ***highly palatable), season (*D* dry season, *R* rainy season, *DR* dry and rainy season)

Only 38.74% of species are available during all seasons (perennial species). Concerning their life form, fodder plants include mostly herbs (58%). These were followed by trees (21%), shrubs (16%) and lianas (5%). The majority of these plants were wild (92%) followed by cultivated (5%) while about 3% were reported as wild or cultivated. Fallows and farmlands (79%) were habitat with high proportion of species. The remaining includes the savannah (16%), forest (3%), habitation and meadow (1% each).

### Plant parts consumed

**Fig. 2:**
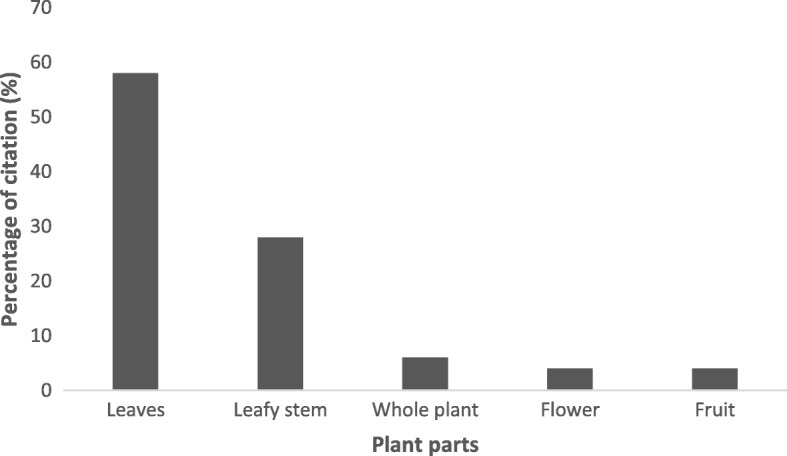
Proportional contributions of plant parts in bovine food diet

Even though major plant parts are significant in the bovine alimentation, leaves were the most commonly used plant part with 58% of citation (Fig. 2). It was followed by leafy stem (28%), flowers and fruits (4% each). However, whole plant was cited in 6% of cases.

### Fodder value about recorded plants

The relative frequencies of citation (RFC) of 116 cited species are shown in Table 3. RFC varies from 1.12 to 5.81%, with 16 species having RFC higher than 1.38 (the average of RFC). Plant species such as *Andropogon gayanus*, *Panicum maximum*, *Pterocarpus erinaceus* and *Flueggea virosa* which were frequently cited were the four dominant plants used as cattle fodder by the breeders in Benin (Table 3). These were followed by *Andropogon tectorum* (RFC = 4.24%), *Anogeissus leiocarpa* (3.16%), *Piliostigma thonningii* (2.83%), *Pseudocedrela kotschyii* (2.57%), *Prosopis africana* (2.52%), *Digitaria horizontalis* (2.29%) and *Annona senegalensis* (2.21%). Those with the lowest citation frequencies included fodder plants such as *Bambusa vulgaris* and *Vetivera nigritana* (0.12% each).

Percentage of fodder value during pasture walk (FVPW) varied from 3% (52 species) to 50% (2 species) (Table 3). We established 3 groups according to the palatability of fodder: 16 highly palatable, 73 weakly palatable fodder and 113 fairly palatable plants (Table 3).

### Selection of priority fodder plants consumed by cattle and their characteristics in Benin

**Fig. 3:**
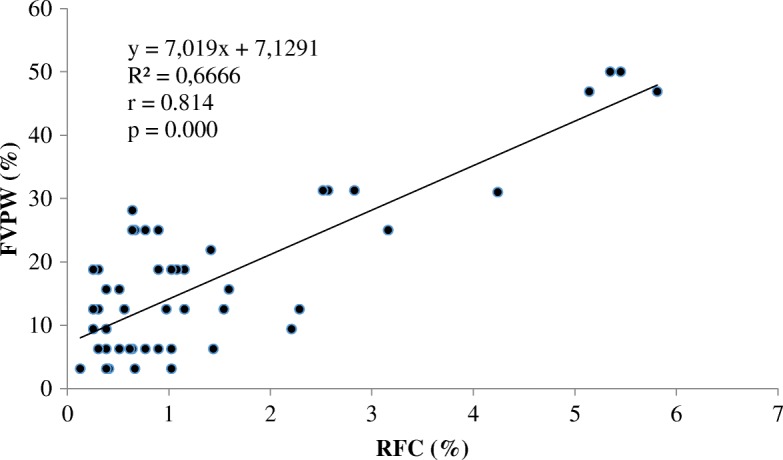
Correlation between relative frequency of citation (RFC) and fodder value during pasture walk (FVPW)

Results from regression analysis showed a significantly positive correlation between relative citation of the species (RFC) and fodder value percentage during pasture walk (FVPW) (*r* = 0.814; *p* < 0.001). There was 66.66% of the variation of RFC that were explained by the variation of FVPW (Fig. 3). Species with higher RFC values often had higher FVPW and included *Andropogon gayanus*, *Panicum maximum* and *Pterocarpus erinaceus*.

We considered the 16 fodder plants having RFC higher than 1.38% (the average of RFC), as top fodder species in Benin (Table 4). According to local people, only 38% of them were highly palatable (Table 4).

**Table 4:** Top 16 fodder plants consumed by the cattle in Benin

N°	Species	Family	RFC	FVPW	P	Ls	MT	PP	Properties
1	*Andropogon gayanus*	Poaceae	5.81	47	**	Ann	Herb	Le	Very good forage
2	*Panicum maximum*	Poaceae	5.45	50	***	Ann	Herb	Le	Good forage
3	*Pterocarpus erinaceus*	Leguminosae	5.34	50	***	Per	Tree	Le	Most consumed in drought, increases weight gain
4	*Flueggea virosa*	Euphorbiaceae	5.14	47	***	Ann	Bushy shrub	LS	Great appetency in drought
5	*Andropogon tectorum*	Poaceae	4.24	31	**	Ann	Herb	Le	Very good forage
6	*Anogeissus leiocarpa*	Combretaceae	3.16	25	**	Per	Tree	Le	–
7	*Piliostigma thonningii*	Leguminosae	2.82	31	**	Per	Tree	Le, Fr	Good appetency
8	*Pseudocedrela kotschyii*	Meliaceae	2.57	31	**	Per	Tree	Le	–
9	*Prosopis africana*	Leguminosae	2.52	31	***	Per	Tree	Le, Fl	Induces milk production
10	*Digitaria horizontalis*	Poaceae	2.28	13	**	Ann	Herb	Le	Good forage
11	*Annona senegalensis*	Annonaceae	2.21	9	*	Per	Shrub	Le	–
12	*Afzelia africana*	Leguminosae	1.59	16	***	Per	Herb	Le	Induces milk production
13	*Acacia sieberiana*	Leguminosae	1.54	13	**	Per	Tree	Le, Fr	Great appetency in drought
14	*Tephrosia purpurea*	Leguminosae	1.54	13	**	Ann	Herb	LS	Anthelmintic
15	*Detarium microcarpum*	Leguminosae	1.44	6	**	Per	Tree	LS	Treat diarrhoea, constipation
16	*Leucaena leucocephala*	Leguminosae	1.41	22	***	Per	Tree	Le	Nutritious plant

*RFC* relative frequency of citation, *FVPW* fodder value during pasture walk, *P* palatability (*fairly, **weakly, ***highly), *Ls* lifespan, *Per* perennial, *Ann* annual, *MT* morphological type, *PP* plant parts used, *Le* leaves, *Fl* flower, *LS* leafed stem, *Fr* fruit

## Discussion

### Diversity of recorded fodder species

Fodder plants consumed by cattle represent 9.01% of the flora of Benin reported by Akoègninou et al. [18]. Among them, only 23.23% are hold by breeders. This shows their low knowledge level about fodder resources. Locally, the clear distinction between the species harvested on pasture and those quoted by the breeders can be explained by the non-control of the plants by the breeders. In vegetation, they are not concerned about feeding cattle as the resource is available and do not continuously monitor the animals. Except in drought, due to lack of grasses, breeders make the choice to cut the branches of shrubs and trees to allow the animals to feed. This was the same on the farms where the drovers cut branches of species to facilitate grazing on the herd. Complementation of cattle diet in the dry season with woody leaves is a common practice in several tropical countries [25–30]. This technique makes it possible to provide supplements and to limit the decline in milk production, but the choice of a well-browsed and productive species is necessary [28]. Among species affected by this practice are *Khaya senegalensis*, *Afzelia africana*, *Prosopis africana*, *Pterocarpus erinaceus*, *Leucaena leucocephala*, *Piliostigma thonningii*, *Acacia sieberiana*, etc. The nutrient input of ligneous fodder is significant in quantitative terms, for reducing seasonal fodder shortfalls and maintaining the livestock, but it is not enough to significantly improve the nitrogen levels of diets, which is a production-limiting factor [29].

Specific richness obtained was 5.27, 10.12 and 1.70 times higher the numbers reported by Sèwadé et al., Sidi et al. and Sinsin et al. [15, 16, 31] respectively for fodder flora in the country. These differences would be due to the national scope of the present study and the combined effect of ethnobotanical studies and the transit walks, contrary to earlier work which covered only part of the country, the ethnobotanical investigations or based only on tree fodder inventory. On the other hand, if we compare our data with the number of fodder species reported outside Benin, specific richness appeared to be relatively higher or lower. César and Zoumana [32] reported 214 species consumed by cattle, sheeps and goats in savannahs of Côte-d’Ivoire. In southwest China [13] and northeast Brazil [6], it was respectively reported 143 and 136 fodder plant species consumed for cattle. These gaps can only be explained by the same arguments given above. Many of these plant species were widely exploited by livestock in other regions of Africa, for example Uganda, Kenya, Zimbabwe, Ethiopia, Nigeria, Rwanda and Mozambique [7, 33–39], and elsewhere in the world [6, 40]. They are species with important nutritious value for ruminants and highly used in cropping systems. We can cite *Leucaena leucocephala*, *Panicum maximum*, *Andropogon gayanus*, *Imperata cylindrica*, *Pterocarpus erinaceus*, *Cynodon dactylon*, *Digitaria horizontalis*, *Anacardium occidentale*, *Mangifera indica*, *Anogeissus leiocarpus*, *Alchornea cordifolia*, *Chamaecrista rotundifolia*, *Eleusine indica*, etc.

Among 185 plant families represented in Benin [18], 29.18% were recorded as fodder plant families. The most diversified in terms of species were Leguminosae and Poaceae. The importance of these families is not a particularity for the fodder flora, but it is a general characteristic of Benin flora because they respectively represent 14.8 and 9.3% among 2807 species [18]. Our findings suggested high genera diversity among recorded species. Thus, in a context of the species rarity, Benin flora provides the possibility to select a great number of fodder species.

### Knowledge about recorded fodder species and use priority by local communities

Though the importance of Leguminosae and Poaceae among recorded plant families is related to the characteristic of Benin flora, this is prominent in the literature, and information regarding the potential productivity and nutritional value is abundant, mainly due to the preference of animals for these two families. Breeders, in permanent touch with their animals, accumulate concurrently day by day the experiences as well on zoo-technique plan as sanitary in order to improve their knowledge on the production and reproduction of animals. Thus, traditional knowledge about fodders of communities should build on the base of their observations and this is orally handed down through generations. Today, they have increased their knowledge and they select great fodders following two main criteria namely quality and availability during the dry season. When we asked factors determining fodder quality, they had cited the palatability, aptitude of the fodder to increase milk production, to treat cattle pathologies, and their ability to fatten cattle. As overall objective of breeders is to sustainably feed cattle in order to improve their production and reproduction, important fodders were selected on the base of these criteria. Indeed, our study revealed Benin breeders preferentially use 16 fodder species that should be considered as priorities. They mostly belong to Leguminosae and Poaceae; Leguminosae being classified as sweet and fattening plants while Poaceae classified as palatable and productive in other regions. These findings are consistent with many studies [9, 41–43]. Among the 16 priority species selected, some have already been identified by Sidi et al. [15] as priority fodder plants in northern Benin namely *Pterocarpus erinaceus*, *Afzelia africana*, *Acacia sieberiana*, *Piliostigma thonningii* and *Flueggea virosa*. These species were also reported in other regions (Sénégal, Cameroon, Niger, etc.) [25, 27, 28] as priority woody species used by pastoralists in Sudanian zone.

Trees and shrubs represented high proportion among fodders cited by local communities. The preference of breeders for these life forms should be due to their availability in all the seasons but also to the relative low contents of crude protein and some minerals in tropical grass species [6, 32, 44, 45].

The plant part used in animal feed is an important criterion of the nutritional [12, 46] and ecological [47] point of view. The widespread use of leaves for fodder in our study is in accordance with the findings of Ayantundé et al. [48] in southwestern Niger, where leaves are the most widely plant part used for fodder and traditional medicine by the agropastoralists.

### Fodder species and sustainable production of cattle in Benin

We think that the valorization and sustainable utilisation of 16 priority fodders could help to improve the cattle production. Among these plants, breeders listed *Afzelia africana*, *Acacia sieberiana*, *Prosopis africana*, *Piliostigma thonningii*, *Digitaria horizontalis*, *Leucaena leucocephala*, *Pterocarpus erinaceus*, *Flueggea virosa*, *Panicum maximum* and *Andropogon gayanus* as forage providing important nutritional properties with high palatability. Literature informs that this nutritive value hold by these plants is due to their content in total nitrogenous substances, which are mostly important in *L. leucocephala*, *P. erinaceus*, *A. africana*, *A. sieberiana*, *P. africana* [48] and *P. maximum* and *A. gayanus* [49]. This makes these plants genuine protein banks for feeding of ruminants during the both seasons due to the presence of two types of fodders (annual and perennial). In addition, according to the breeders, some of these fodders hold many medicinal properties. *Tephrosia purpurea* was recognised as being efficiently used to treat helminthiasis, whereas *Detarium microcarpum* was cited to address several gastrointestinal disorders notably diarrhoea and constipation. Furthermore, breeders recognised *P. africana* and *A. africana* as plants involved in increasing of the production of milk after their grazing by the cow. This knowledge hold by local breeders comes from a deep relation between human and biological resources of its local environment. Volpato and Puri [49] showed the Sahrawi recognise in detail the relations between forage and the taste, smell or health and nutritional properties of camel milk because camel milk was the main output of camel husbandry and a staple food in the Sahrawi pastoral system. Currently, the valorization of the local knowledge related to these species needs further studies in particular phytochemical and pharmacological to confirm medicinal properties, as well as anatomical, to identify their anti-nutritional drivers’ content such as lignins, which block the digestibility of nitrogen in rumen.

Most of top fodders form a component of livelihood strategies in the country because they remain an important source of health care and constitute an essential basis in traditional medicine improvement. They are also valued for their timber and their trade importance. Unfortunately, the large combined and increasing demand for these plants and the consequent increase in the rate of collection negatively affected the wild populations of many species, to the point that some species are now considered to be threatened with extinction. Thus, 2 fodder species among 16 priorities (12.50%) were classified as endangered plant species according to the International Union for Nature Conservation (https://www.iucnredlist.org/) and Adomou et al. [5]. We will cite *A. africana* and *P. erinaceus*. This handicaps their sustainable use. Agroforestry species such as *Vitellaria paradoxa* and *Khaya senegalensis* benefit from particular management practices such as assisted natural regeneration, seeding or often sapling transplantation within the farmlands [50]. But some species as *A. africana* seems to be neglected [50]. Urgent conservation measures must be taken for ensuring their sustainability use in Benin.

Pasture production is traditionally unknown in Benin, but forage cultivation is done on national farms [51]. Cultivated fodders have been experimented with but are of little importance in smallholder stock rearing. Fortunately, some fodders are cropped in several state farms such as *L. leucocephala*, *Brachiaria* spp., *P. maximum* and *A. gayanus.* However, this does not fully ensure their fodder needs for livestock. So the development of a breeding program or improvement of the priority forage species on these farms should be considered. After a promising species has been identified, evaluated and developed into a cultivar by selection or breeding, the seed of the resulting cultivar has to be made available to farmers for testing and use.

## Conclusion

The combination of ethnobotanical studies and transit walks constituted efficient means for the documentation of 257 fodder plants consumed by cattle in Benin. Specific richness obtained during transit walk demonstrates the importance of follow-up in identifying fodder plants. In addition, this paper provided the lifespan, life form, most commonly used parts for fodder, in palatability, status, and a listing of priority fodder plants. The 16 top priorities were considered as important fodder resources used in Benin. Further studies are needed including an anatomical evaluation of 16 fodder species consumed by cattle for assessing their digestive capacity.

## Abbreviations

Ann: Annual
C: Cultivated
D: Dry season
DR: Dry and rainy season
FEB: State farm of Bétécoucou
FEK: State farm of Kpinnou
FEO: State farm of Okpara
FES: State farm of Samiondji
Fl: Flowers
Fr: Fruits
FVPW: Fodder value during pasture walk
Le: Leaves
Leg: Leguminosae
LS: Leafy stems
MT: Morphological type
Per: Perennial
PP: Plant parts
R: Rainy season
RFC: Relative frequency of citation
Temp: Temperature
tub: Tubercle
W: Wild
WC: Wild and cultivated
WP: Whole plant
